# Supplementary material for: Diagnostic Performance of a Molecular Assay in Synovial Fluid Targeting Dominant Prosthetic Joint Infection Pathogens
Source: Microorganisms. 2024 Jun 19;12(6):1234. doi: 10.3390/microorganisms12061234 (PMC11206145; doi:10.3390/microorganisms12061234)
Supplement: Supplementary file 1 [file microorganisms-12-01234-s001.zip › microorganisms-3023239-supplementary.pdf]

Supplementary tables

Table S1. Accession numbers of the selected nine dominant PJI bacteria and four dominant PJI fungi

**Primer-hydrolysis probe set**	**Target**	**Gene**	**Organism**	**Accession no.**	**Last accessed**
Set 1 (DreamDX-bac-primer-hydrolysis probe)	Pan-bacteria (this study)	16S rRNA	*Staphylococcus epidermidis*	NR_036904.1	December 8, 2023
			Staphylococcus aureus	NR_118997.2	December 8, 2023
			Streptococcus dysgalactiae	NR_027517.1	December 8, 2023
			Escherichia coli	NR_024570.1	December 8, 2023
			Acinetobacter baumannii	NR_026206.1	December 8, 2023
			Enterococcus faecalis	MF108410.1	December 8, 2023
			Streptococcus pyogenes	MT535878.1	December 8, 2023
			Pseudomonas aeruginosa	NR_026078.1	December 8, 2023
			Enterobacter cloacae	NR_102794.2	December 8, 2023
Set 2 (DreamDX-fun-primer-hydrolysis probe)	Pan-fungi (this study)	18S rRNA	*Candida glabrata*	JF825465.1	April 18, 2024
			Candida albicans	LC612900.1	April 18, 2024
			Candida tropicalis	KX664670.1	April 18, 2024
			Candida parapsilosis	LC643454.1	April 18, 2024

Table S2. Reference strains used in this study

	Strain designated in the standard	ATCC/KCTC number
Bacterial	*Staphylococcus epidermidis*	ATCC 35989
	Staphylococcus aureus	ATCC 29213
	Enterococcus faecalis	KCTC 3511
	Streptococcus dysgalactiae	KCTC 3098
	Streptococcus pyogenes	ATCC 19615
	Escherichia coli	ATCC 35150
	Acinetobacter baumannii	KCTC 23254
	Pseudomonas aeruginosa	KCTC 22063
	Enterobacter cloacae	ATCC 2361
Fungal	*Candida parapsilosis*	KCTC 7653
	Candida tropicalis	KCTC 7212
	Candida glabrata	KCTC 7219
	Candida albicans	ATCC 10231

Abbreviations: KCTC, Korean Collection for Type Cultures, Taejon, Korea; ATCC, American Type Culture Collection

Table S3. Sequences of universal primers used in this study

Primer	Sequence	Target	Reference
27F	5′-AGAGTTTGATCMTGGCTCAG-3′	Bacteria	[24]
1492R	5’-TACGGYTACCTTGTTACGACTT-3′	Bacteria	[24]
ITS1F	5′-TCCGTAGGTGAACCTGCGG-3′	Fungi	[25]
ITS4R	5′-TCCTCCGCTTATTGATATGC-3′	Fungi	[25]

Abbreviations: F: forward primer, R: reverse primer

Table S4. Microorganism culture and identification

Sample source	No.	Microorganism	Numbers of colonies
Mokdong Himchan Hospital	1	*Staphylococcus aureus* (Bacteria)	numerous
	2	*Staphylococcus aureus* (Bacteria)	numerous
	3	*Streptococcus dysgalactiae* (Bacteria)	numerous
	4	*Escherichia coli* (Bacteria)	moderate
	5	*Staphylococcus epidermidis* (Bacteria)	few
	6	*Staphylococcus epidermidis* (Bacteria)	few
	7	*Serratia marcescens* (Bacteria)	moderate
	8	*Streptococcus dysgalactiae (*Bacteria)	numerous
Incheon Himchan Hospital	9	*Enterobacter aerogenes* (Bacteria)	few

Table S5. 16S rRNA and 18S rRNA target gene sequences

Organisms	Target strain	Target gene sequences of 16S rRNA and 18S rRNA
Bacteria	*Staphylococcus epidermidis*	5′-GAGGAAAGTGGAATTCCATGTGTAGCGGTGAAATGCGCAGAGATATGGAGGAACACCAGTGGCGAAGGCGACTTTCTGGTCTGTAACTGACGCTGATGTGCGAAAGCGTGGGGATCAAACAGGATTAGATACCCTGGTAGTCCACGCCGTAAACGATGAGTGCTAAGTGTTAGGGGGTTTCCGCCCCTTAGTGCTGCAGCTAACGCATTAAGCACTCCGCCTGGGGAGTACGACCGCAAGGTTGAAACTCAAAGGAATTGACGGGGACCCGCACAAGCGGTGGAGCATGTGGTTTAATTCGAAGCAACGCGAAGAACCTT-3′
	Staphylococcus aureus	5′-GAGGAAAGTGGAATTCCATGTGTAGCGGTGAAATGCGCAGAGATATGGAGGAACACCAGTGGCGAAGGCGACTTTCTGGTCTGTAACTGACGCTGATGTGCGAAAGCGTGGGGATCAAACAGGATTAGATACCCTGGTAGTCCACGCCGTAAACGATGAGTGCTAAGTGTTAGGGGGTTTCCGCCCCTTAGTGCTGCAGCTAACGCATTAAGCACTCCGCCTGGGGAGTACGACCGCAAGGTTGAAACTCAAAGGAATTGACGGGGACCCGCACAAGCGGTGGAGCATGTGGTTTAATTCGAAGCAACGCGAAGAACCTT-3′
	Enterococcus faecalis	5′-GAGGAGAGTGGAATTCCATGTGTAGCGGTGAAATGCGTAGATATATGGAGGAACACCAGTGGCGAAGGCGGCTCTCTG

	GTCTGTAACTGACGCTGAGGCTCGAAAGCGTGGGGAGCA AACAGGATTAGATACCCTGGTAGTCCACGCCGTAAACGA TGAGTGCTAAGTGTTGGAGGGTTTCCGCCCTTCAGTGCTG CAGCTAACGCATTAAGCACTCCGCCTGGGGAGTACGACC GCAAGGTTGAAACTCAAAGGAATTGACGGGGGCCCGCAC AAGCGGTGGAGCATGTGGTTTAATTCGAAGCAACACGAA GAACCTT-3′
Streptococcus dysgalactiae	5′- GGGGAGAGTGGAATTCCATGTGTAGCGGTGAAATGCGTA GATATATGGAGGAACACCGGTGGCGAAAGCGGCTCTCTG GTCTGTAACTGACGCTGAGGCTCGAAAGCGTGGGGAGCA AACAGGATTAGATACCCTGGTAGTCCACGCCGTAAACGA TGAGTGCTAGGTGTTAGGCCCTTTCCGGGGCTTAGTGCCG CAGCTAACGCATTAAGCACTCCGCCTGGGGAGTACGACC GCAAGGTTGAAACTCAAAGGAATTGACGGGGGCCCGCAC AAGCGGTGGAGCATGTGGTTTAATTCGAAGCAACGCGAA GAACCTT-3′
Streptococcus pyogenes	5′- GGGGAGAGTGGAATTCCATGTGTAGCGGTGAAATGCGTA GATATATGGAGGAACACCGGTGGCGAAAGCGGCTCTCTG GTCTGTAACTGACGCTGAGGCTCGAAAGCGTGGGGAGCA AACAGGATTAGATACCCTGGTAGTCCACGCCGTAAACGA TGAGTGCTAGGTGTTAGGCCCTTTCCGGGGCTTAGTGCCG GAGCTAACGCATTAAGCACTCCCGCCTGGGGAGTACGAC CGCAAGGTTGAAACTCAAAGGAATTGACGGGGGCCCGCA

	CAAGCGGTGGAGCATGTGGTTTAATTCGAAGCAACGCGA<br>AGAACCTT-3′
Escherichia coli	5′-<br>GAGGGGGGTAGAATTCCAGGTGTAGCGGTGAAATGCGTA<br>GAGATCTGGAGGAATACCGGTGGCGAAGGCGGCCCCCTG<br>GACGAAGACTGACGCTCAGGTGCGAAAGCGTGGGGAGCA<br>AACAGGATTAGATACCCTGGTAGTCCACGCCGTAAACGA<br>TGTCGACTTGGAGGTTGTGCCCTTGAGGCGTGGCTTCCGG<br>AATTAACGCGTTAAGTCGACCGCCTGGGGAGTACGGCCG<br>CAAGGTTAAAACTCAAATGAATTGACGGGGGCCGCACAA<br>GCGGTGGAGCATGTGGTTTAATTCGATGCAACGCGAAGA<br>ACCTT-3′
Acinetobacter baumannii	5′-<br>GAGGATGGTAGAATTCCAGGTGTAGCGGTGAAATGCGTA<br>GAGATCTGGAGGAATACCGATGGCGAAGGCAGCCATCTG<br>GCCTAATACTGACGCTGAGGTACGAAAGCATGGGGAGCA<br>AACAGGATTAGATACCCTGGTAGTCCATGCCGTAAACGA<br>TGTCTACTAGCCGTTGGGGCCTTTGAGGCTTTAGTGGCGC<br>AGCTAACGCGATAAGTAGACCGCCTGGGGAGTACGGTCG<br>CAAGACTAAAACTCAAATGAATTGACGGGGGCCCGCACA<br>AGCGGTGGAGCATGTGGTTTAATTCGATGCAACGCGAAG<br>AACCTT-3′
Pseudomonas aeruginosa	5′-<br>GAGGGTGGTGGAATTTCCTGTGTAGCGGTGAAATGCGTA<br>GATATAGGAAGGAACACCAGTGGCGAAGGCGACCACCTG

		GACTGATACTGACACTGAGGTGCGAAAGCGTGGGGAGCA AACAGGATTAGATACCCTGGTAGTCCACGCCGTAAACGA TGTCGACTAGCCGTTGGGATCCTTGAGATCTTAGTGGCGC AGCTAACGCGATAAGTCGACCGCCTGGGGAGTACGGCCG CAAGGTTAAAACTCAAATGAATTGACGGGGGCCCGCACA AGCGGTGGAGCATGTGGTTTAATTCGAAGCAACGCGAAG AACCTT-3′
	Enterobacter cloacae	5′- GAGGGGGGTAGAATTCCAGGTGTAGCGGTGAAATGCGTA GAGATCTGGAGGAATACCGGTGGCGAAGGCGGCCCCCTG GACAAAGACTGACGCTCAGGTGCGAAAGCGTGGGGAGCA AACAGGATTAGATACCCTGGTAGTCCACGCCGTAAACGA TGTCGACTTGGAGGTTGTGCCCTTGAGGCGTGGCTTCCGG AGCTAACGCGTTAAGTCGACCGCCTGGGGAGTACGGCCG CAAGGTTAAAACTCAAATGAATTGACGGGGGCCCGCACA AGCGGTGGAGCATGTGGTTTAATTCGATGCAACGCGAAG AACCTT-3′
Fungi	*Candida glabrata*	5′- TTTTAGTAGAAAACAACTTCAAAACTTTCAACAATGGATC TCTTGGTTCTCGCATCGATGAAGAACGCAGCGAAATGCG ATACGTAATGTGAATTGCAGAATTCCGTGAATCATCGAAT CTTTGAACGCACATTGCGCCCTCTGGTATTCCGGGGGGCA TGCCTGTTTGAGCGTCATTTCCTTCTCAAACACGTT-3′
	Candida albicans	5′- CAGATTATTACTTAATAGTCAAAACTTTCAACAACGGATC

	TCTTGGTTCTCGCATCGATGAAGAACGCAGCGAAATGCG ATACGTAATATGAATTGCAGATATTCGTGAATCATCGAAT CTTTGAACGCACATTGCGCCCTCTGGTATTCCGGAGGGCA TGCCTGTTTGAGCGTCGTTTCTCCCTCAAACCGCTG-3′
Candida tropicalis	5′-GATTTATTATTACAATAGTCAAAACTTTCAACAACGGATC TCTTGGTTCTCGCATCGATGAAGAACGCAGCGAAATGCG ATACGTAATATGAATTGCAGATATTCGTGAATCATCGAAT CTTTGAACGCACATTGCGCCCTTTGGTATTCCAAAGGGCA TGCCTGTTTGAGCGTCATTTCTCCCTCAAACCCCCG-3′
Candida parapsilosis	5′-GATTATATTTATTAATAGTCAAAACTTTCAACAACGGATC TCTTGGTTCTCGCATCGATGAAGAACGCAGCGAAATGCG ATAAGTAATATGAATTGCAGATATTCGTGAATCATCGAAT CTTTGAACGCACATTGCGCCCTCTGGTATTCCGGAGGGCA TGCCTGTTTGAGCGTCATTTCTCCCTCAAACCCCCG-3′

Table S6. Serum TP level, CPR, whole-blood ESR and WBC count, and synovial fluid WBC count in the culture-positive and -negative groups

		Clinical laboratory test range	Culture result	
			Positive (n = 9)	Negative (n = 84)
Serum	TP (g/dL)	5.8–8.1	6.7 ± 0.83	7.0 ± 0.61
	CRP (mg/dL)	0.0–0.3	12.4 ± 9.36	5.2 ± 6.62
Whole blood	ESR (mm/h)	0–9	66.7 ± 25.81	36.0 ± 36.83
	WBC (10^3 cells/μL)	4.23–9.07	12.0 ± 5.46	8.3 ± 2.99
Synovial fluid	WBC	Normal: < 200<br>Non-inflammatory: 200–2,000<br>Inflammatory: 2,000–10,000	33520.0 ± 3733.52	1285.0 ± 9388.73

Septic: > 80,000

Hemorrhagic: 200–2,000

Abbreviations: TP, total protein; CPR, C-reactive protein; ESR, erythrocyte sedimentation rate; WBC, white blood cell

Data are mean ± standard deviation
